# New Normal: Emergence of Situational Leadership During COVID-19 and Its Impact on Work Motivation and Job Satisfaction

**DOI:** 10.3389/fpsyg.2022.919941

**Published:** 2022-06-21

**Authors:** Sarfraz Aslam, Atif Saleem, Tribhuwan Kumar, Khalida Parveen

**Affiliations:** ^1^School of Foreign Languages, Yulin University, Yulin, China; ^2^College of Teacher Education, College of Education and Human Development, Zhejiang Normal University, Jinhua, China; ^3^Department of English Language and Literature, College of Science and Humanities at Sulail, Prince Sattam Bin Abdulaziz University, Al Kharj, Saudi Arabia; ^4^Faculty of Education, Southwest University, Chongqing, China

**Keywords:** New Normal, situational leadership, COVID-19, work motivation, job satisfaction

## Introduction

Globally, COVID-19 has caused rapid changes in the workplace (Kirby, 2020). COVID-19 has disrupted the standard working order of all organizations, including educational, health, business, etc. This has affected workers' motivation and job satisfaction. Suffering and challenges reduce workers' happiness and productivity (Singh and Mishra, 2020). Motivation at work is an essential criterion for a healthy organization, particularly in an epidemic context (Wang et al., 2021). We need to employ new leadership behaviors that harness uncertainty to improve employee motivation and job satisfaction. This article provides theoretical support and practical reference for organizations to cultivate situational leadership and eliminate employees' exhaustion to improve work motivation and job satisfaction.

## COVID-19 and Leadership

COVID-19 has affected governments globally, and societies are experiencing an odd situation; after the global pandemic, this situation led to a global crisis that touched the aspect of our lives, including family, education, health, work, and the relationship between leaders and followers in our society (Hinojosa et al., 2020; Aslam et al., 2021; Parveen et al., 2022a). Organization leaders play a critical role in framing employee experiences at the workplace during and after the pandemic as they adapt to work on new realities (Ngoma et al., 2021). The managerial level of communication of those who lead still has a substantial impact on their followers' performance, behavior, and mental health (Wu and Parker, 2017; Saleem et al., 2020; Parveen et al., 2022b).

## New Normal

“New Normal” has been used since the end of World War II (Francisco and Nuqui, 2020). An indispensable leader knows how to do ordinary things well; an unafraid leader acts regardless of criticism and never backs down (Honore and Robinson, 2012). Nevertheless, the new normal in 2020 is different since the COVID-19 pandemic has affected the world's economy and education. This is an uphill battle in which education and money are at stake in a situation where people find it challenging to adjust. This shift in working and learning space is defined as the New Normal in working organizations (Mollenkopf et al., 2020). It is moving from a public to a private space, shifting from one-size-fits-all methods to individualized and differentiated learning, shifting responsibility. Active participation of household members is required for this learning process and for evaluating learning shifts (Francisco and Nuqui, 2020).

Herein, in this study, we examine: how organizations attain excellent performance in the face of the COVID-19 pandemic through a situational leadership approach?

The human resource department is one of the most important aspects of any organization. Organizations, irrespective of their form and goals, are based on various visions for the benefit of humans. Additionally, the process is by implementing its mission and is handled by humans. To achieve performance superiority, any organization should concentrate on brilliant employees. The impact of globalization on knowledge and technology progress in many different areas is incomprehensible. It is indispensable for the management of human resources to be among the most critical organizational assets since it plays a significant role in developing and achieving organization objectives (Syaifuddin and Sidu, 2019).

## Social Exchange Theory (SET)

Organizational behavior theories such as SET (Blau, 1964) are the most influential approach (Cropanzano and Mitchell, 2005). According to SET (Gouldner, 1960), a good deed performed by a leader engenders positive behaviors by the opposite party (a subordinate). Leaders who serve as role models are likely to feel obligated to their duties and show greater interest in their assigned tasks (Liborius, 2014). Using the social exchange perspective, employees whose leaders encourage them through participative leadership behaviors, such as participation in decision-making and increased responsibility, may thrive more and offer helpful behavior toward coworkers due to this increased autonomy (Usman et al., 2021).

## Nature of Situational Leadership

Leadership style is a person's approach to influencing others through their behavior pattern. The directive, as well as supportive behavior, compose this leadership style. A directive behavior encourages group members to achieve goals by providing direction, setting goals and providing evaluation methods, defining roles, assigning deadlines, showing how they will accomplish the objectives, and establishing timelines, which are spelled out, often through one-way communication. Group members who exhibit supportive behaviors are more likely to feel comfortable in their group, coworkers, and situation. Social and emotional support is demonstrated through supportive behaviors; supportive behaviors demand two-way communication (Northouse, 2021). Providing direction, implementing and monitoring plans, and motivating team members are aspects of a leadership style (Hourston, 2013). An organization administrator capable of adapting to the current circumstances is situational leadership.

Through a situational approach, followers advance and regress in a developmental continuum that measures the relative competence and commitment of the followers. Leaders must determine where followers are on the developmental continuum to adapt their leadership style accordingly (Northouse, 2021). Situational leadership is characterized by the relation between the task behavior (giving instructions, directing, guiding, and valuing) and the listening, supporting, and valuing aspects of the engagement. Combined strategies that consider individuals and the environment are advantageous for this style. Consequently, workers can maximize their learning experiences and satisfaction (Walls, 2019). In following a situational leader, it is not as necessary to have a charismatic leader with large numbers of followers as it is to have rational comprehension of the situation and appropriate response (Grint, 2011). Situational leadership requires individuals to be flexible and use their behavior according to their situation without following a set formula (Walls, 2019).

## Work Motivation

Motivation determines what individuals do and how they do it based on what they are motivated to do (Meyer et al., 2004). Motivating someone to act to achieve his or her goals is a condition or circumstance that encourages and stimulates a person. As a result of solid motivation, an individual may possess energy, power, or a complex condition and the ability to move toward a particular goal, whether or not it is achieved. The motivation will be driven by both the individual (intrinsic) and his surroundings (extrinsic). According to Herzberg's theory, a motivational factor would be achievement, recognition, responsibility, progress, the work itself, and the opportunity to develop. Work motivation factors include achievement, recognition, and advancement (Syaifuddin and Sidu, 2019).

## Job Satisfaction

The sense of comfort and pride employees experience in doing their jobs is called job satisfaction; job satisfaction is achieved by employees who feel their job is valuable and essential (Mustofa and Muafi, 2021). The belief in the amount of pay employees must get for the differences in rewards becomes a general attitude toward their work assessment (Castle et al., 2007). Besides, job satisfaction is related to what they get and expect (Dartey-Baah and Ampofo, 2016). Then, it will be represented by positive or negative behavior that employees showed in the workplace (Adiguzel et al., 2020). Several factors have influenced job satisfaction, including working hours, working conditions, payment, work design, promotions, demographic features, human resource development, leadership style, and stress level (Bhardwaj et al., 2021). There is a direct correlation between job satisfaction and an organization's leadership style that provides advice, praise, and assistance to employees when they face problems at work (Sapada et al., 2017; Phuc et al., 2021). Employees who are highly satisfied with their job can contribute to the organization's performance (Takdir et al., 2020). Employees often focus less on the duties and responsibilities of an employee than on perceived job satisfaction that encourages them to perform at their best (Aprilda et al., 2019).

## Relationship of Situational Leadership With Work Motivation and Job Satisfaction

There is a positive correlation between work motivation and job satisfaction, and intrinsic motivation is positively correlated with job satisfaction (Alnlaclk and Alnlaclk, 2012). In research, it was discovered that intrinsic motivation was positively related to job satisfaction (Arasli et al., 2014).

Leadership and work motivation provide a positive and significant effect on job satisfaction (Pancasila et al., 2020). Leadership motivates and satisfies followers by helping them in a friendly way (Haq et al., 2022). According to several studies, situational leadership leads to increased motivation (Fikri et al., 2021). Situational leadership can positively and significantly affect job satisfaction and trust, respect, and pride among subordinates. Incorporating these characteristics can assist leaders in building employee commitment, raising risk awareness, articulating a shared vision, and reinforcing the importance of the vision (Al-edenat, 2018). The result is also in line with that of Li and Yuan (2017), who demonstrated that a leader's impact on job satisfaction is both positive and significant. According to Saleem (2015), leadership creates a significant positive impact on job satisfaction. Situational leadership is positively associated with job satisfaction (Fonda, 2015). In conclusion, leadership is crucial in determining work motivation and job satisfaction (Mustofa and Muafi, 2021).

## Summary and Conclusions

**Figure 1:**
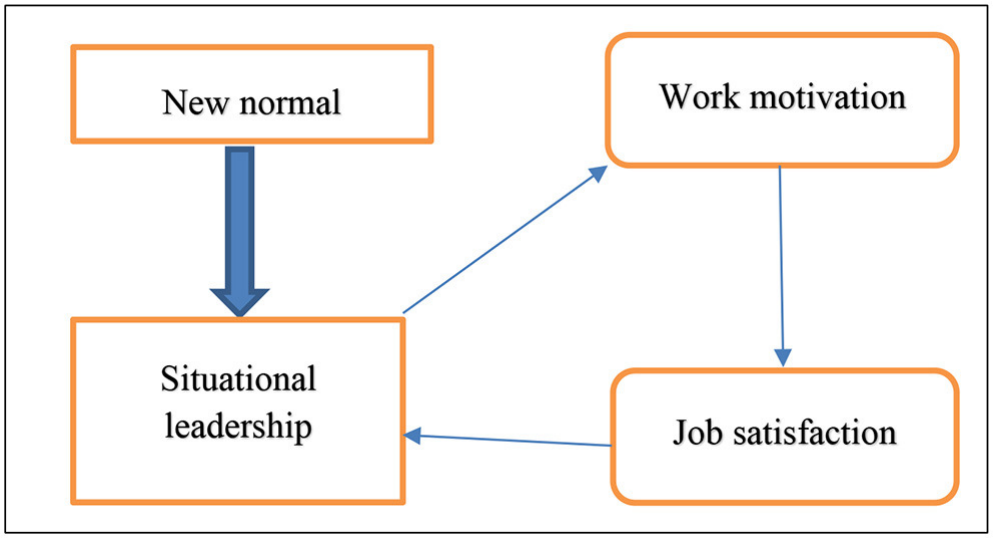
Conceptual model.

Situational leadership has a positive influence on work motivation and job satisfaction (Figure 1). It encourages employees to finish their jobs enthusiastically and spurs their devotion to their roles for successful job completion. This leadership style is easy to comprehend, intuitively sensible, and adaptable to various situations (Northouse, 2021).

Situational leadership significantly impacts job satisfaction (Shyji and Santhiyavalli, 2014). Assuring employee job satisfaction is a vital role of a leader in achieving organizational goals. Job satisfaction levels may vary between employees, places, jobs, and organizations (Ridlwan et al., 2021; Saleem et al., 2021). In addition to promoting exemplary employees, effective leadership promotes job satisfaction (Setyorini et al., 2018). Employee job satisfaction directly impacts job performance in an organization (Hutabarat, 2015). Employee performance is positively correlated with job satisfaction (Sidabutar et al., 2020). This situational approach has a prescriptive component, whereas many leadership theories are descriptive. Situational leadership, for instance, prescribes a directing style for you, the leader, if your followers are of very low competence. The situational approach suggests that you follow a supportive leadership style if your followers appear competent but lack confidence. These prescriptions, in general, provide all leaders with a set of guidelines that are extremely helpful for aiding and enhancing effective and efficient leadership (Northouse, 2021).

Leaders should be aware of how they lead and use appropriate styles to develop the skills of their staff while promoting satisfaction with their jobs (Carlos do Rego Furtado et al., 2011).

In sum, situational leadership motivates employees and improves employee satisfaction at work (Schweikle, 2014). The situational approach applies to virtually any organization and at nearly any level for almost any goal. There are many possible applications for it (Northouse, 2021). Higher productivity resulted from better leadership. In this way, job satisfaction contributes to employee performance ultimately. That means the higher job satisfaction leads to the better the employee performance (Jalagat, 2016). Effective leadership can result in more satisfied employees, more motivation at work, and more satisfaction with the workplace. It is worth mentioning that the theoretical understandings gained through this research will encourage future scholars to investigate how situational leaders can improve the performance of employees. An extensive empirical study is needed to understand the role of the situational leadership approach in the current pandemic circumstances. Moreover, the biggest challenge facing leadership studies right now is the lack of knowledge about the topic.

## Author Contributions

SA presented the main idea and wrote the first draft of the manuscript. AS contributed to revising and proofreading the manuscript. After review, TK and KP helped us finalize the revisions and proofreading. All authors contributed to the article and approved the submitted version.

## Conflict of Interest

The authors declare that the research was conducted in the absence of any commercial or financial relationships that could be construed as a potential conflict of interest.

## Publisher's Note

All claims expressed in this article are solely those of the authors and do not necessarily represent those of their affiliated organizations, or those of the publisher, the editors and the reviewers. Any product that may be evaluated in this article, or claim that may be made by its manufacturer, is not guaranteed or endorsed by the publisher.
